# Intragastric pH of foals admitted to the intensive care unit

**DOI:** 10.1111/jvim.15888

**Published:** 2020-09-29

**Authors:** Jessica C. Wise, Sharanne L. Raidal, Edwina J. A. Wilkes, Kristopher J. Hughes

**Affiliations:** ^1^ Veterinary Clinical Centre, School of Animal and Veterinary Science Charles Sturt University Wagga Wagga New South Wales Australia

**Keywords:** foals, horse, omeprazole, ulcers

## Abstract

**Background:**

Intragastric pH profiles of neonatal foals admitted to the intensive care unit (ICU) remain poorly characterized.

**Hypothesis/Objectives:**

To determine intragastric pH profiles and clinical parameters associated with intragastric pH in foals admitted to the ICU.

**Animals:**

Forty‐two neonatal foals admitted to the ICU and requiring placement of an indwelling nasogastric tube for nutritional management were included.

**Methods:**

Intragastric pH was measured for 24 hours from the time of admission. Mean pH, % time pH <4, and % time pH >4 were determined for each foal. History, clinical findings, and clinicopathological data recorded at the time of presentation were collected.

**Results:**

The mean pH of included foals was 5.5 ± 1.8. The median % time pH <4 was 6.3% (range: 0‐99). A history of placentitis was associated with greater mean pH (median 5.3 (range: 0.9‐7.8) versus median 7.2 (5.9‐11.3); *P* = .002) and less % time pH <4 (median 13 (0‐99.6) versus median 0.1 (0‐7.2); *P* = .01). Foals with diarrhea had a greater % time pH <4 (median 4.6% (0‐99) versus median 28.8% (1.4‐57.48); *P* = .02). Foals with a pH >4 for >50% recording time had a lower PaO_2_ (mean difference 25.0 mm Hg; 95% confidence interval [CI], 14.4‐35.6; *P* = .03) and higher PaCO_2_ (mean difference 14.9 mm Hg; 95% CI, 4.7‐25.2; *P* = .02). Surviving foals had a lower mean median hourly pH (*P = .*02).

**Conclusions and Clinical Importance:**

Intragastric pH profiles were unpredictable and mostly >4 for >80% of the recording time. This study does not support the indiscriminate administration of acid suppressive treatment.

**Abbreviations**
% time pH >4: percentage of time intragastric pH >4
95% CI: 95% confidence interval
AUC: area under the gastric pH‐time curve
EGUS: equine gastric ulcer syndrome
ESGD: equine squamous gastric disease
ICU: intensive care unit
NGT: nasogastric tube
OR: odds ratio
pH >4_>50_: pH >4 for >50% of the recording period
pH >4_>80_: pH >4 for >80% of the recording period

## 1 INTRODUCTION

Gastric ulceration is an important condition of foals, presenting as 4 clinical syndromes: subclinical disease, clinical disease, gastric perforation, and pyloric stricture.^1^ The prevalence of this condition in foals is reported to be up to 57%,^2^ with a prevalence of 22% in nonsurviving foals hospitalized between birth and 6 months of age.^3^ Affected neonatal foals rarely demonstrate the clinical signs observed in older foals.^4^

Due to the reported high prevalence of gastric lesions in neonatal foals,^2^, ^3^, ^5^ and concern for potential fatal consequences of gastric ulceration, neonatal foals in intensive care unit (ICU) settings are frequently administered acid‐suppressive medication empirically, without confirmation of gastric ulceration,^6^ or measurement of gastric pH. However, recent studies suggest the prevalence of gastric ulceration is lower in neonates than older foals,^3^ and the intragastric pH profiles and response to medical suppression of gastric acid secretion in critically ill foals might differ from healthy foals.^7^ Gastric acid might be beneficial, providing an important barrier against bacterial colonization of the intestinal tract in foals, as has been described for human infants,^8^ and the production of gastric acid is associated with survival in clinically ill neonatal foals.^4^ Administration of acid‐suppressive medications to neonatal foals admitted to the ICU is associated with increased risk of undifferentiated diarrhea during hospitalization,^6^ further questioning the practice of empirical administration of these therapeutics.

Pharmacodynamic targets for prevention or treatment of equine gastric ulcer syndrome (EGUS) and acid suppressive treatment remain ill‐defined. In humans, the healing of gastroesophageal reflux disease, which is a similar disease process to equine squamous gastric disease (ESGD), is achieved when the time pH is >4 exceeds 66%.^9^ The results of previous studies suggest that increasing the intragastric pH so that the percentage of time pH <4 was limited to no more than 20 to 50% facilitated the healing of EGUS,^10^, ^11^, ^12^, ^13^ and prevention of ESGD has been demonstrated when intragastric pH was >4 for 49% of time.^10^

The intragastric pH profiles of hospitalized equine neonates remain poorly characterized, including the likelihood of sustained periods of pH <4. As such, the prognostic importance of intragastric pH, factors that are associated with gastric acid secretion, and justification for acid suppressive therapy in sick foals are unknown. The aims of this study were: (1) to determine the intragastric pH profiles of systemically ill foals admitted to the neonatal ICU and (2) to identify clinical and clinicopathological parameters associated with intragastric pH <4.

## 2 MATERIALS AND METHODS

The study population comprised of systemically ill neonatal foals, <14 days of age, admitted to the ICU at the Veterinary Clinical Centre, Charles Sturt University, between August 2017 and February 2018. All foals included in the study required placement of an indwelling nasogastric tube (NGT) for nutritional management. Prior to placement, a calibrated disposable antimony pH probe with 2 electrodes 5 cm apart (Greenfield Single use pH catheter with 2 pH antimony channels, 5 cm spacing 6.4 French diameter, internal reference; product number Medical Management Systems (MMS)‐pH‐5; via MD Solutions Pty Ltd, Williamstown North, Victoria) was placed within the lumen of an NGT (Dover Flush Enema Tube, Ref 155733, Covidien Lane Cove Australia), as part of the hospital standard operating procedures (Supplementary Item 1). The pH probe was secured with the most proximal pH electrode at the end of the NGT. The NGT was passed and secured at the level of nares by a suture. The NGT and pH electrode were considered in place when gastric fluid could be aspirated through the tube, or abdominal radiography confirmed the NGT and pH probe within the gastric lumen. The pH probe was connected to a data logger (Ohmega Ambulatory pH Recorder, MD Solutions Pty Ltd, Williamstown North, Australia) that was secured to the foal's neck by a bandage. The data recording system recorded pH from both electrodes every second. Continuous measurement of intragastric pH was scheduled for 24 hours from the time of admission for each foal. Electrode placement did not interfere with feeding or reflux of milk through the NGT. At the end of the recording period, the electrode was removed without dislodging the NGT. If the foal required the indwelling NGT be retained for enteric nutrition supplementation at 24 hours after the removal of the initial pH electrode, another calibrated pH electrode was placed within the lumen of the NGT and another 24 hours of recording was attempted. Endoscopic examination of the gastric mucosa was not routinely performed.

Signalment (foal age, breed, and sex) and clinical data including primary disease process, gestation length, history/evidence of placentitis, body weight at presentation, ambulatory or recumbency status, feeding schedule, results of hematological and blood biochemical examinations, bacterial culture of blood, and acid‐base parameters of arterial blood recorded at the time of presentation were collected for each foal.

### 2.1 Data analysis

**FIGURE 1:**
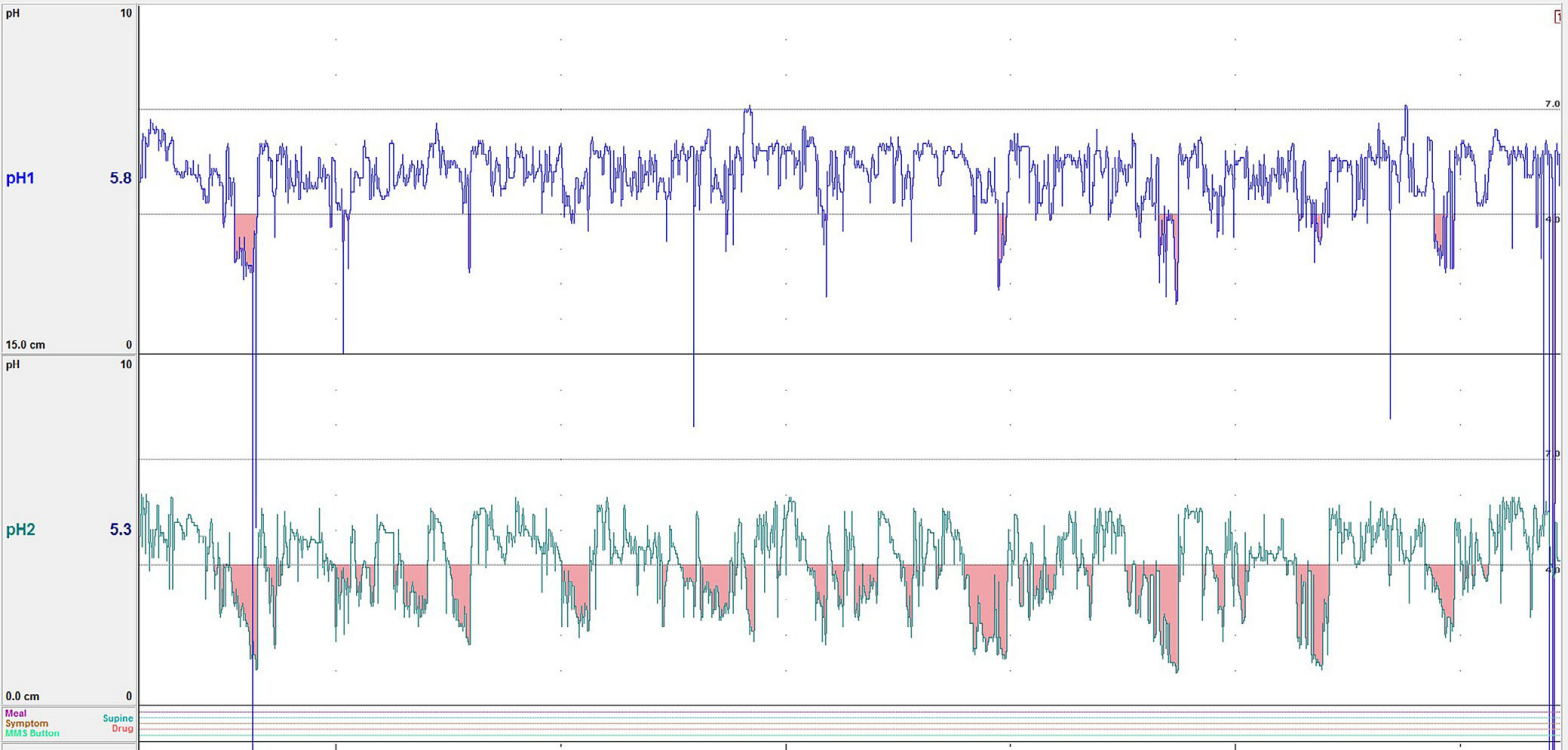
Example of the graph of pH over time for the proximal (top) and distal (bottom) pH electrodes provided by the Medical Management Systems (MMS) software, with pH <4 highlighted by red shading

When recording was finished, the data were imported from the data logger onto a personal computer using commercially available software (MMS database software; Version 9.5, February 23, 2017; http://ww.mmsinternational.com/) and graphs of pH over time, including periods of pH <4 were created to visually assess pH profiles (Figure 1). Data were exported to Microsoft Excel for calculation of mean pH, median hourly pH, area under the time‐median hourly pH curve (AUC), % time pH <4, % time pH >4 and the duration of the recording for each foal. The number of foals with pH >4 for >50% (pH >4_>50_) and for >80% (pH >4_>80_) of the recording time were determined.

Statistical analysis was performed using R (version 2.6‐1, 2019‐11‐19). A Shapiro‐Wilk test was performed to differentiate parametric and nonparametric data. Parametric data are presented as mean ± SD and nonparametric data are presented as median (range). Differences in the data collected from the distal pH probe and the proximal pH probe were investigated compared by paired *t* tests (parametric data) and Wilcoxon signed‐rank test (nonparametric data). Outcome variables, analyzed independently for proximal and distal electrodes, were median hourly pH, mean pH and % time pH <4 as continuous variables, and pH >4_>50_ and pH >4_>80_ as dichotomous variables. Independent *t* tests (parametric data) and Mann‐Whitney *U* tests (nonparametric data) were used to assess for differences in pH between groups based on clinical variables, including disease status (yes/no). Categorical data were compared to the dichotomous outcomes pH >4_>50_ and pH >4_>80_ using Fisher's exact tests. Continuous variables were compared with the dichotomous outcomes of pH >4_>50_ and pH >4_>80_ by univariate logistic regression. All variables with *P* value >.2 were included in multivariable logistic regression with backwards stepwise removal of variables and inclusion of interactions. Median hourly pH were compared between surviving and nonsurviving foals using a linear mixed effects model with data fitted using restricted maximum likelihood and with the Geisser‐Greenhouse correction to accommodate unequal sphericity; post hoc comparisons were performed using the Sidak method. The AUC was determined for each foal using the trapezoid rule, and results compared between surviving and nonsurviving foals by independent *t* tests. For all analyses, significance was set at *P* < .05.

In foals where a second recording period occurred, differences between the 24 hours of recording from the time of admission, and recording from 48 hours from admission were compared using dependent *t* tests (parametric data) and Wilcoxon signed‐rank test (nonparametric data).

## 3 RESULTS

### 3.1 Foals

Forty‐two foals were included in the study (Supplementary Item 2): 27 (64%) colts and 15 (36%) fillies. The study population included predominantly Thoroughbreds (n = 17, 40%) and Standardbreds (n = 13, 31%), but also included Quarter horses (n = 3), Arabians (n = 3), American Paint (n = 3), and heavy Draught breeds (n = 3). The mean gestational length of the foals was 341 ± 15.2 days. Four foals were premature with a gestational age of less than 320 days. The median age at the time of presentation was 31 hours (range: 2 hours to 11 days). Thirty‐one foals were less than 24 hours of age at the time of presentation. Twenty‐eight (67%) foals were ambulatory at the time of presentation and 19 (45%) foals had nursed from the mare. Primary diagnoses included neonatal multisystemic maladjustment syndrome (n = 29), diarrhea (infectious and noninfectious diarrhea) (n = 11) (5 foals overlapped both categories), musculoskeletal problems (n = 2), neonatal isoerythrolysis (n = 1), and uroperitoneum (n = 1). Three foals presented with sick mares or as orphan foals.

Nine of 30 foals that had blood collected for microbiological examination had a positive blood bacterial culture. Hematological and blood biochemical examinations were performed in 40 foals, of which 15 (38%) had leukogram changes and/or increased concentrations of acute phase proteins, consistent with inflammation. All foals had semiquantitative measurement of IgG, and 28 foals had inadequate passive transfer of immunoglobulins (IgG <8 g/L). Twenty foals (48%) were hypoxemic, using age‐dependent reference ranges (PaO_2_ < 70 mm Hg), and 15 of these foals demonstrated hypoventilation (PaCO_2_ > 50 mm Hg). Eleven foals (26%) were acidemic (peripheral blood pH of <7.35). Two foals demonstrated net gastric reflux at the time of presentation. Thirty‐four foals (81%) were discharged from hospital, 7 (17%) foals were subjected to euthanasia, and 1 (2%) foal died (Supplementary Item 2).

### 3.2 Treatment

Thirty‐five (83%) foals were administered resuscitative fluid therapy administered intravenously at the time of presentation, and subsequently received fluid therapy administered intravenously at maintenance rates. Twenty‐five (60%) foals received a constant rate infusion of glucose administered intravenously. Intranasal insufflation of oxygen was administered to 24 (57%) foals. Antimicrobial drugs were administered systemically to 37 (88%) foals. Other treatment administered to individual foals included NSAIDs, thiamine, frusemide, hyperimmune plasma transfusion, topical ophthalmic treatments, respiratory stimulants, and vasopressors. Mare's milk was administered via the indwelling NGT according to nutritional requirements and tolerance of enteral feeding. If the foal demonstrated signs of abdominal discomfort or gastric reflux, feeding was discontinued. No foal in the current study received gastric acid suppressive treatment at the time of intragastric pH recording.

### 3.3 pH data

**FIGURE 2:**
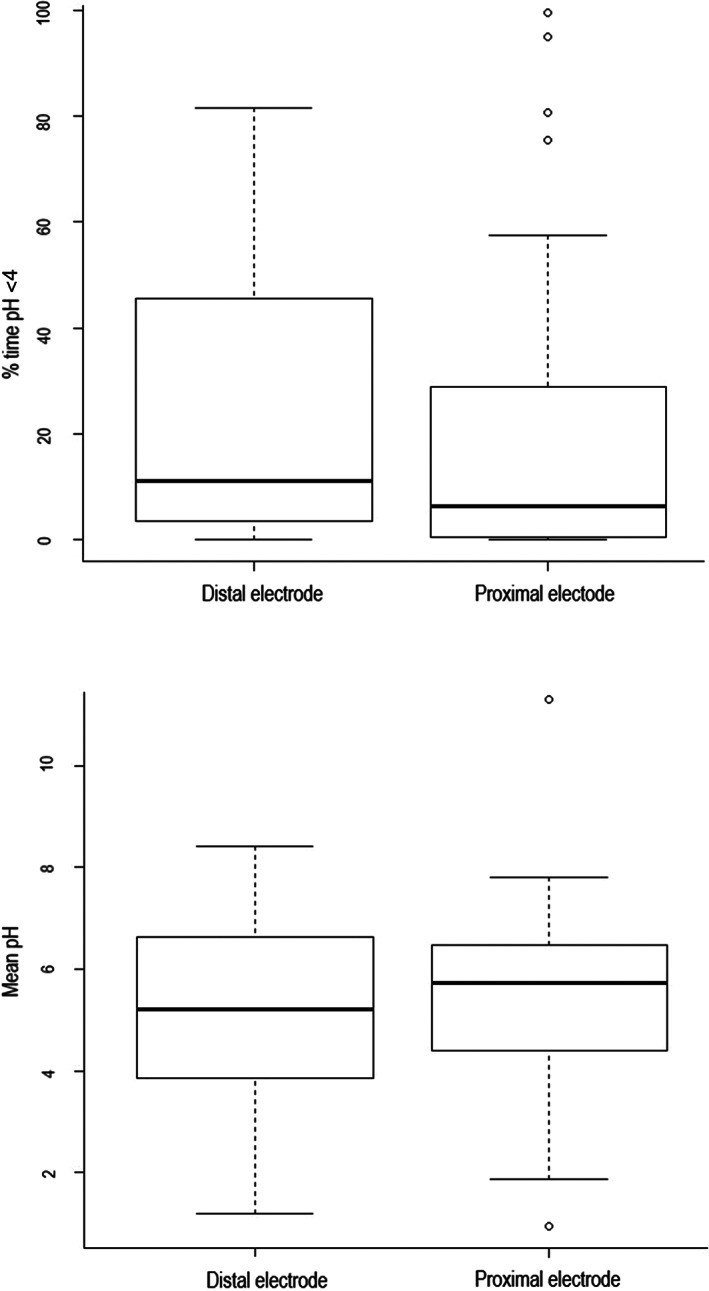
Box and whisker plot comparing the proximal and distal electrodes in the first recording period. There was a difference in mean pH (*P* = .03) and no difference in % time pH <4. The median is marked by the horizontal line, the box spans the interquartile range and the whiskers span the upper and lower limits. The outliers, calculated by Tukey's rule, are indicated by circles

Twenty‐four hours of recording was not achieved in all foals for various reasons including probe damage or breakage and malfunction of the data logger. The mean duration of pH recording was 18.7 ± 10.6 hours (Supplementary Item 3). Fourteen foals had a pH recording of 24 hours or longer. There was no difference in % time pH <4 between the proximal electrode and the distal electrode (*P* = .07), while there was a difference in mean pH (*P* = .03) (Figure 2). There was no observed diurnal variation in intragastric pH.

### 3.4 Proximal electrode

The mean of the mean pH of all 42 foals was 5.5 ± 1.8. Five foals had a mean intragastric pH <4 and 34 foals had a mean pH <7. All foals had a pH measurement <7 at some point during the recording, and in 3 foals, the intragastric pH did not decrease below 4 at any time during the recording. The median percentage of time that pH was <4 was 6.3% (range: 0‐99). Thirty‐seven foals (88%) had intragastric pH >4_>50_ (mean recording time 19 ± 10 hours; mean of mean pH 6 ± 1.4) and 29 foals (69%) had a pH >4_>80_ (mean recording time 18 ± 10 hours, mean of mean pH 6.4 ± 1.2).

### 3.5 Distal electrode

The mean of the mean pH of all foals was 5.2 ± 1.8. Eleven foals had a mean pH <4 and 34 foals had a mean pH <7. All foals had a pH measurement <7 at some point during the recording, and in 3 foals, the intragastric pH did not decrease below 4 at any time during the recording. The median percentage of time that pH was <4 was 11% (range: 0‐82%). Thirty‐two foals (76%) had a pH >4_>50_ (mean recording time 17.3 ± 10.2 hours; mean of mean pH 5.8 ± 1.5) and 25 foals (60%) had a pH >4_>80_ (mean recording time 16.8 ± 11 hours; mean of mean pH 6.2 ± 1.5).

### 3.6 Clinical associations

**FIGURE 3:**
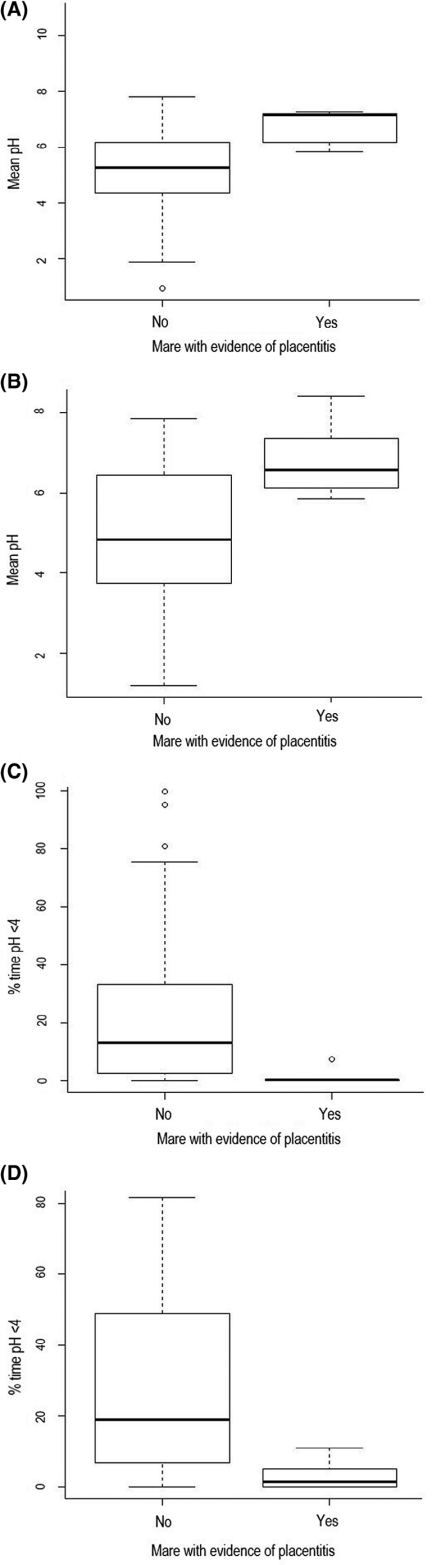
Box and whisker plots demonstrating the mean pH (A and B) and % time pH <4 (C and D) for foals presenting with mare with (n = 35) or without (n = 7) a history of placentitis. Data are presented for both the proximal electrode (A and C) and distal electrode (B and D). A history of placentitis is associated with an increased mean pH (*P* = .002) and decreased % time pH <4 (*P* = .01). The median is marked by the horizontal line, the box spans the interquartile range and the whiskers span the upper and lower limits. The outliers, calculated by Tukey's rule, are indicated by circles

**FIGURE 4:**
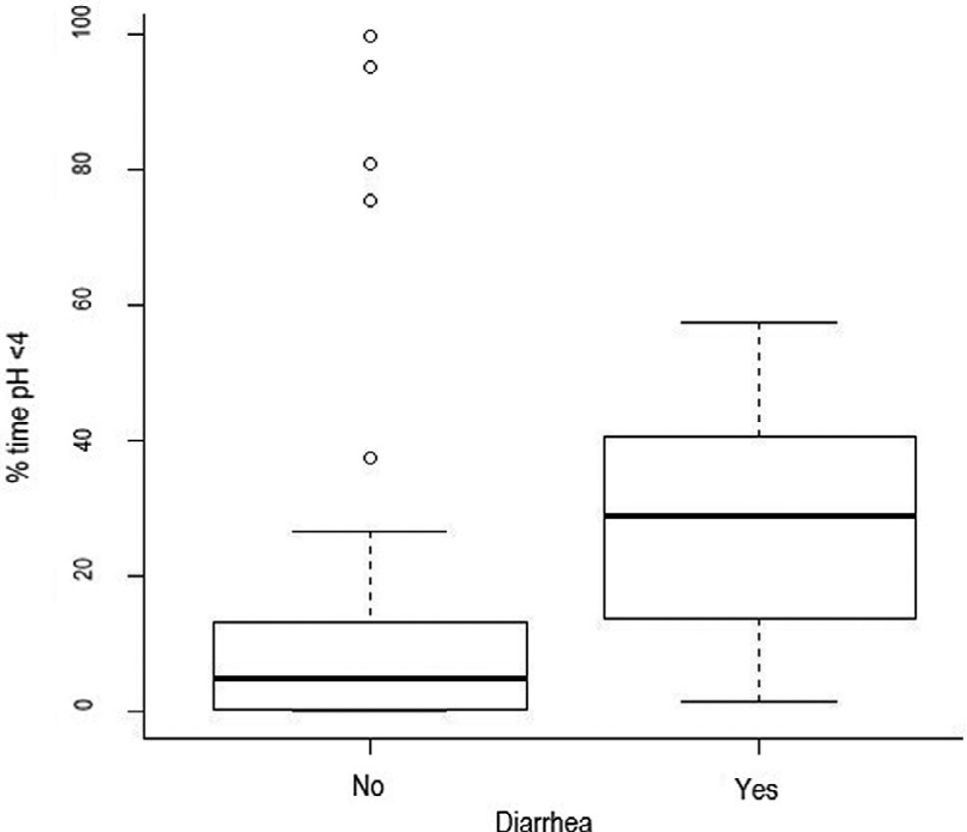
Box and whisker plot of the difference in % time pH <4 for foals admitted to the ICU with diarrhea (n = 11) and without diarrhea (n = 31) (*P* = .02). The median is marked by the horizontal line, the box spans the interquartile range and the whiskers span the upper and lower limits. The outliers, calculated by Tukey's rule, are indicated by the circles

**FIGURE 5:**
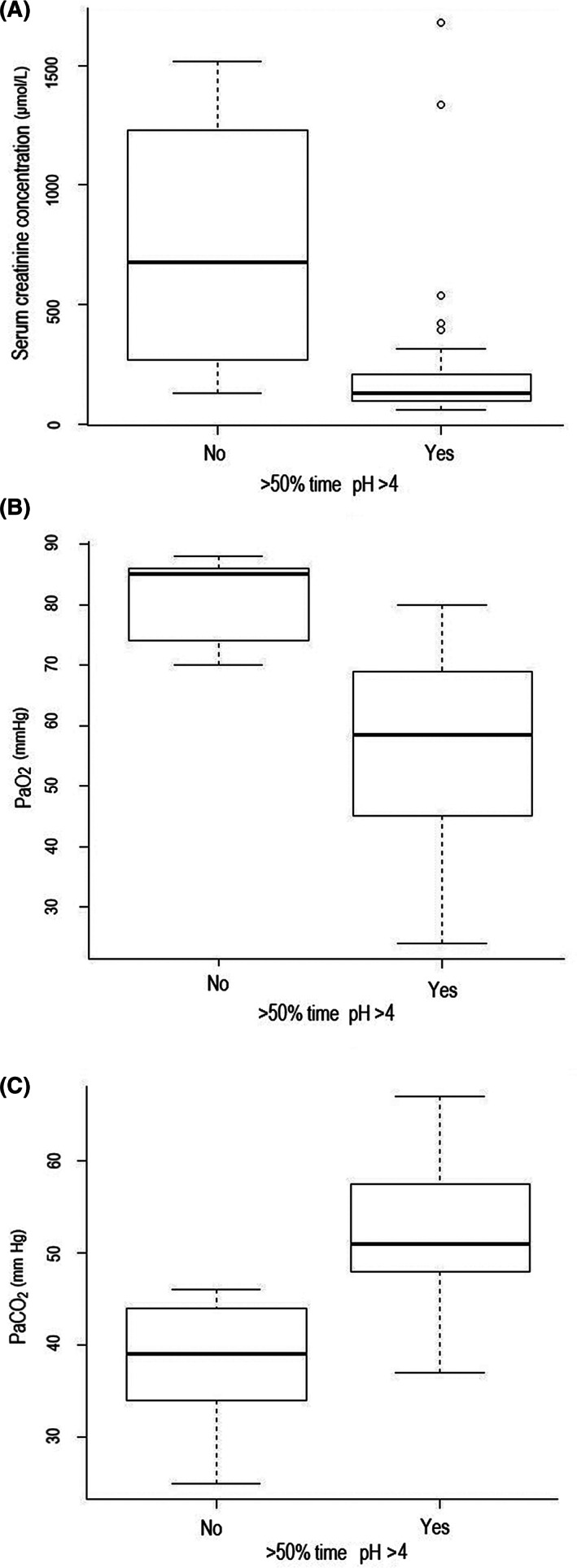
Box and whisker plots of differences in serum creatinine concentration (A) (*P* = .04), PaO_2_ (B) (*P* = .03), and PaCO_2_ (C) (*P* = .02) for foals that did (n = 37) or did not (n = 5) have an intragastric pH >4 for >50% of recording time with the distal electrode. The median is marked by the horizontal line, the box spans the interquartile range and the whiskers span the upper and lower limits. The outliers, calculated by Tukey's rule, are indicated by circles

A history/evidence of placentitis was associated with a greater mean pH (*P* = .002) and lower % time pH <4 (*P* = .01) (Figure 3; Supplementary Item 4). Conversely, foals that presented with diarrhea had a greater % time pH <4 in comparison to foals without diarrhea, when measuring pH with the proximal electrode (*P* = .02) (Figure 4; Supplementary Item 4), and were less likely to have a pH >4_>80_ when measuring with the proximal electrode (*P* = .02, Supplementary Item 5). Foals with a more alkaline intragastric pH profile, evidenced by pH >4_>50_, had a lower serum creatinine concentration (*P* = .04), higher PaCO2 (*P* = .02) and lower PaO_2_ (*P* = .03, Figure 5; Supplementary Item 6). In all multivariable models, no parameters were retained at the multivariable level.

**FIGURE 6:**
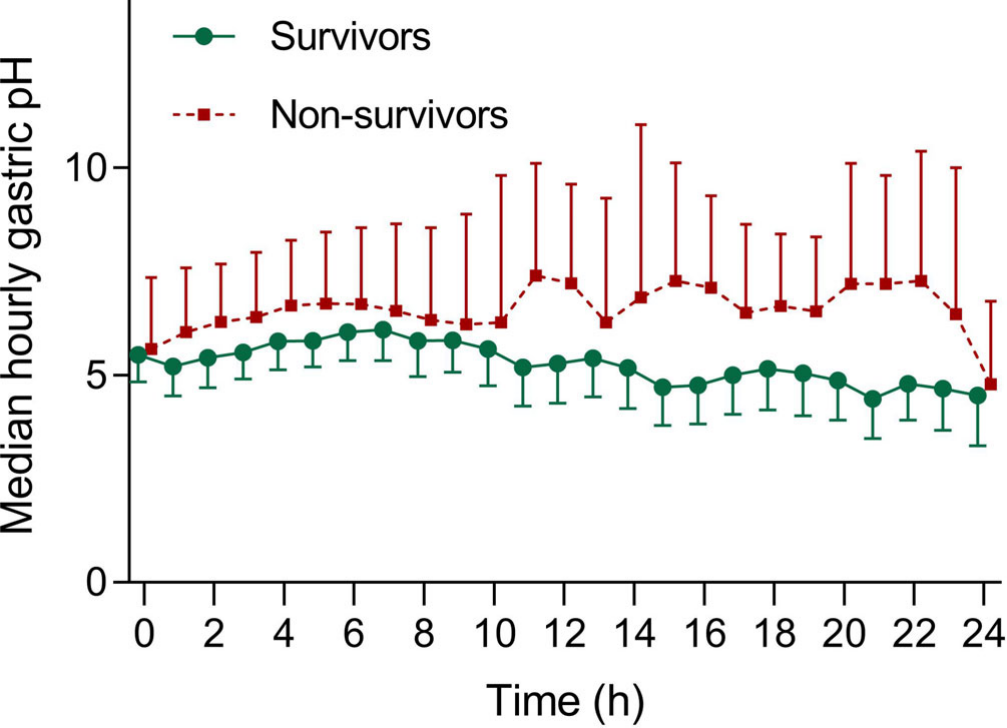
Mean and 95% confidence interval (CI) of the median hourly pH of foals that survived to discharge (n = 34) or were nonsurvivors (subjected to euthanasia or died; n = 8). Surviving foals had a lower median hourly pH for the duration of recording (*P* = .02); post hoc comparisons were not different at any time point. Data are shown as mean and 95% CI

Overall, for the proximal electrode mean median hourly pH for the duration of the recording (*P* = .02, Figure 6) was lower in surviving foals than in foals that were subjected to euthanasia or died. The AUC was lower in surviving foals (mean difference 36.1; 95% confidence interval [CI], 0.4‐71.8; *P* = .05). There was no difference in mean pH, % time pH <4 between surviving foals and nonsurviving foals.

### 3.7 Second recording period

Eleven foals had a second intragastric pH recording beginning 24 hours after completion of the first recording period. There was no difference between the mean pH or % time pH <4 of the first 24 hours of recording and the second 24 hours of recording (Supplementary Item 7). Mean pH was 5.5 ± 2.3, and % time pH <4 was 15% (range: 0‐64). Seven foals (64%) foals had a pH >4_>80_ in the second recording period. Nine foals (82%) had a pH >4_>50_ in the second recording period.

## 4 DISCUSSION

This study revealed that in the majority (69%) of foals presented to the ICU, intragastric pH was >4 for >80% of the recording period and in and 88% of foals, the intragastric pH was >4 for at least 50% of the recording period. This finding supports a previous report that acid suppressive therapy in foals admitted to the ICU might not be necessary due to the tendency for foals to have an intragastric pH >4 for protracted periods.^4^ In humans, gastric pH studies demonstrate that gastro‐esophageal reflux disease resolves when gastric pH is >4 for over 60% of the time.^9^, ^14^ Given the role of gastric acidity in both human gastro‐esophageal reflux disease and ESGD, it can be inferred that ulceration of the squamous mucosa in horses is unlikely to occur when intragastric pH is greater than 4 for over 80% of the time^12^, ^15^, ^16^ as was observed for most foals included in this study.

In our hospital population, and as previously reported,^4^ foals that survived to discharge had a lower mean median hourly pH for the duration of the recording and lower AUC when compared to foals that were subjected to euthanasia or died. However, within this study, no difference in mean pH, % time pH <4 was observed between foals that survived to discharge and foals that were subjected to euthanasia or died. The high survival rate in the current study (81%) could have influenced the results of hypothesis testing of associations between pH and survival. The ability of surviving foals to secrete gastric acid is likely associated with improved tissue perfusion and organ function, or disease of lesser severity. Alternatively, hospital practices for nutritional support of foals might include reduction of the frequency of feeding and increasing the volume of milk provided at each feed, as neonates respond to treatment. Consequently, increased gastric acidity might be associated with feeding practices in surviving neonates. Conversely, critically ill neonates were more likely to have increased gastric alkalinity, consistent with impaired gastric secretory function or reduced parasympathetic tone and resultant reduction in parietal cell stimulation. The increased gastric alkalinity might also be attributed to gastric dysmotility^17^ and enterogastric reflux. The results within this study do not support the ability to predict survival based on measurement of intragastric pH measurement, and further investigation into the relationship between survival and intragastric pH is warranted.

There was no apparent difference between intragastric pH profiles at the time of admission when compared with profiles obtained after 48 hours of treatment, consistent with previous studies demonstrating that intragastric pH profiles in foals change minimally in the first week of life.^18^ As such, protracted measurement of intragastric pH for periods of greater than 24 hours is unnecessary when assessing pH profiles and need for acid‐suppressive treatment in foals.

A history of placentitis and evidence of respiratory insufficiency (decreased PaO_2_ and increased PaCO_2_) were associated with increased intragastric pH. These findings are suggestive of decreased parietal cell function and indicate that, in foals that were unable to secrete gastric acid, gastric mucosal function or integrity was compromised by marked pre‐ or peripartum disease. In support of our findings, in humans and guinea pigs, the secretion of HCl represents normal physiological function of parietal cells, and is compromised by hypoxia, endotoxin and critical illness.^19^ Abnormal gastric motility, leading to increased enterogastric reflux^17^, might also impact intragastric pH in these foals.

Foals that had a presenting complaint of diarrhea or had evidence of diarrhea at the time of presentation had more acidic intragastric pH profiles. This association was only apparent in the proximal electrode, and with % time pH >4. Reasons for an association between a lower intragastric pH in foal with diarrhea were not determined in our study. Gastric acidity has previously been suggested to be a protective mechanism against the development of diarrhea secondary to bacterial colonization of the gastrointestinal tract.^8^ However, in a previous study of hospitalized foals, gastric ulceration was positively associated with other forms of gastrointestinal disease,^3^ and the authors speculated that ulceration might develop due to gastric acidity in addition to fluctuations in fluid and electrolyte balance, abnormal intestinal motility, intestinal inflammation, and visceral pain. While it is possible that the foals with diarrhea in the current study were at increased risk of gastric injury, given the acidic intragastric pH profiles, examination of the gastric mucosa was not undertaken. The intragastric pH profiles of the foals prior to the development of diarrhea is unknown, and further investigation into the relationship between intragastric pH and diarrhea in neonatal foals is required.

In the current study, increased concentration of creatinine in plasma was associated with decreased gastric acidity, suggesting that parietal cell function was preserved in the face of renal or prerenal azotaemia, or placental insufficiency, in these foals.

This study focused on the intragastric pH profile of critically ill foals, rather than the effect of treatment. Previous studies have investigated the effects of ranitidine or omeprazole on intragastric pH of small groups of clinically healthy or hospitalized foals.^4^, ^7^, ^20^ In the current study, the mean hourly pH ranged from 0.9 to 11 and percentage of recording time pH was less than 4 ranged from 0 to 99%. Similarly, considerable differences in pH profiles between foals were reported in a previous study of critically ill neonates.^4^ These results indicate that foals are capable of marked variation in intragastric pH; however, the association of pH and risk of gastric ulceration remains uncertain. In the current study, while the relationship between intragastric pH and the prevalence of gastric ulcerative disease within neonatal foals admitted to the ICU was not explored, gastric ulceration was not observed in any of the nonsurviving foals at necropsy. Although larger than previous studies of gastric pH in hospitalized foals,^4^, ^7^ the number of foals in the current study was modest, which might have influenced the outcomes of the multivariable models used to explore associations between clinical and clinicopathological associations and intragastric pH. Other limitations of the current study include the wide variation in gestation, the broad spectrum of disease presentation, and treatment regimens between foals. The methodology of intragastric pH measurement used in this study was adapted from previously reported methods used in both adult horses and neonatal foals.^4^, ^7^, ^12^, ^18^ Although the placement of the probe within the NGT and the confirmation of location within the gastric lumen were confirmed for each foal in the study, factors such as patient death, probe breakage or malfunction of the data logger precluded 24 hours of continuous pH recording for some foals. The anatomical location of pH probes, although confirmed within the gastric lumen, could not be precisely controlled in recumbent patients at all times. Changes in intragastric pH were considered to reflect parietal cells function; however enterogastric reflux and difference in the position of electrodes within the stratification of the pH of gastric contents could also impact intragastric pH recorded using this methodology. Further studies on the intragastric pH and associations with clinical parameters and survival and the prevalence and consequences of gastric ulceration in foals are warranted.

## 5 CONCLUSION

The majority of the foals included within this study had an intragastric pH of >4 for >80% of the recording time, which does not support the indiscriminate use of acid‐suppressive medications in critically ill foals. Our results suggest that nonsurviving foals, foals with a history of placentitis, and foals presenting with respiratory insufficiency, had higher intragastric pH. Conversely, foals presenting with diarrhea had more acidic intragastric profiles. The intragastric pH profiles of critically ill neonatal foals admitted to the ICU were variable and further research is required to better understand the clinical significance of different pH measurements.

## CONFLICT OF INTEREST DECLARATION

Kristopher J. Hughes serves as Associate Editor for the Journal of Veterinary Internal Medicine. He was not involved in review of this manuscript.

### OFF‐LABEL ANTIMICROBIAL DECLARATION

Authors declare no off‐label use of antimicrobials.

### INSTITUTIONAL ANIMAL CARE AND USE COMMITTEE (IACUC) OR OTHER APPROVAL DECLARATION

Authors declare no IACUC or other approval was needed.

### HUMAN ETHICS APPROVAL DECLARATION

Authors declare human ethics approval was not needed for this study.

## Supporting information


**Supplementary Item 1** Photograph of the indwelling nasogastric tube, with the calibrated disposable antimony pH probe with 2 electrodes 5 cm apart, seated within the lumen. The pH probe is secured at the most proximal end of the nasogastric tube. The pH probe is connected to a data logger that was secured to the foal's neck by a bandage.

**Supplementary Item 2** Clinical data collected at the time of admission from 42 foals presented to the ICU that underwent measurement of intragastric pH.

**Supplementary Item 3** Intragastric pH data collected from 42 foals that were presented to the ICU.

**Supplementary Item 4** Results of univariate analyses of associations between dichotomised clinical parameters (yes/no) and the outcome variables of mean pH and % time < pH 4, for both the proximal and distal electrodes.

**Supplementary Item 5** Results of univariate analyses of associations between clinical parameters (yes/no) and pH > 4 for >50% of the recording period (pH >4_>50_) and pH >4 for >80% of the recording period (pH >4_>80_) as dichotomous variables (yes/no) for both the proximal and distal electrodes.

**Supplementary Item 6** Results of univariate logistic regression analyses of associations between continuous clinical parameters and the outcome variables pH >4 for >50% of the recording period (pH >4_>50_) and pH >4 for >80% of the recording period (pH >4_>80_).

**Supplementary Item 7** Comparison of the distal electrode in the first recording period to the second recording period. There is no significant difference in mean pH or % time pH < 4 in the 2 recording periods.

## References

[1] Andrews FM , Nadeau JA . Clinical syndromes of gastric ulceration in foals and mature horses. Equine Vet J. 1999;31:30‐33. 10.1111/j.2042-3306.1999.tb05165.x 10696290

[2] Murray MJ , Murray CM , Sweeney HJ . Prevalence of gastric lesions in foals without signs of gastric disease: an endoscopic survey. Equine Vet J. 1990;22:6‐8. 2298194 10.1111/j.2042-3306.1990.tb04193.x

[3] Elfenbein JR , Sanchez LC . Prevalence of gastric and duodenal ulceration in 691 nonsurviving foals (1995‐2006). Equine Vet J. 2012;41:76‐79. 10.1111/j.2042-3306.2011.00449.x 22594031

[4] Sanchez LC , Lester GD , Merritt AM . Intragastric pH in critically ill neonatal foals and the effect of ranitidine. J Am Vet Med Ass. 2001;218:907‐911. 11294316 10.2460/javma.2001.218.907

[5] Barr BS , Wilkins P , Del Piero F , et al. Is prophylaxis for gastric ulcers necessary in critically ill equine neonates? A retrospective study of necropsy cases 1989‐1999. J Vet Intern Med. 2000;14:328.

[6] Furr M , Cohen ND , Axon JE , et al. Treatment with histamine‐type 2 receptor antagonists and omeprazole increase the risk of diarrhoea in neonatal foals treated in intensive care units. Equine Vet J. 2012;44:80‐86. 10.1111/j.2042-3306.2011.00499.x 22594032

[7] Javsicas LH , Sanchez LC . The effect of omeprazole paste on intragastric pH in clinically ill neonatal foals. Equine Vet J. 2008;40:41‐44. 18083658 10.2746/042516407X235803

[8] Dinsmore JE , Jackson RJ , Smith SD . The protective role of gastric acidity in neonatal bacterial translocation. J of Ped Surg. 1997;32:1014‐1016. 10.1016/s0022-3468(97)90389-4 9247224

[9] Bell NJV , Dw B , Howden C , et al. Appropriate acid suppression for the management of gastro‐oesophageal reflux disease. Digestion. 1992;51:59‐67. 1397746 10.1159/000200917

[10] McClure SR , White GW , Sifferman RL , et al. Efficacy of omeprazole paste for prevention of recurrence of gastric ulcers in horses in race training. J Am Vet Med Assoc. 2005;226:1685‐1688. 15906569 10.2460/javma.2005.226.1685

[11] Sykes BW , Underwood C , Greer R , McGowan CM , Mills PC . The effects of dose and diet on the pharmacodynamics of omeprazole in the horse. Equine Vet J. 2017;49:525‐531. 27554924 10.1111/evj.12630

[12] Raidal SL , Andrews FM , Nielsen SG , Trope G . Pharmacokinetic and pharmacodynamic effects of 2 omeprazole formulations on stomach pH and gastric ulcer scores. Equine Vet J. 2017;49:802‐809. 28432741 10.1111/evj.12691

[13] Merritt AM , Sanchez LC , Burrow JA , Church M , Ludzia S . Effect of GastroGard and three compounded oral omeprazole preparations on 24 h intragastric pH in gastrically cannulated mature horses. Equine Vet J. 2003;35:691‐695. 14649361 10.2746/042516403775696339

[14] Hunt RH . The relationship between the control of pH and healing and symptom relief in gastro‐oesophageal reflux disease. Aliment Pharmacol Ther. 1995;9:3‐7. 10.1111/j.1365-2036.1995.tb00777.x 7495939

[15] Sykes BW , Underwood C , McGowan CM , et al. The effects of dose and diet on the pharmacokinetics of omeprazole in the horse. J Vet Pharmacol Ther. 2017;40:172‐178. 27478135 10.1111/jvp.12345

[16] McClure SR , White GW , Sifferman RL , et al. Efficacy of omeprazole paste for prevention of gastric ulcers in horses in race training. J Am Vet Med Assoc. 2005;226:1681‐1684. 15906568 10.2460/javma.2005.226.1681

[17] Hess‐Dudan F , Rossdale PD . Neonatal maladjustment syndrome and other neurological signs in the newborn foal: part 1. Equine Vet Educ. 1996;8:24‐32.

[18] Sanchez LC , Lester GD , Merritt AM . Effect of ranitidine on intragastric pH in clinically normal neonatal foals. J Am Vet Med Assoc. 1998;212:1407‐1412. 9589127

[19] Modlin IM , Basson MD , Adrian TE , et al. Pepsinogen release and acid secretion from human and Guinea pig gastric mucosa compromised by hypoxia, endotoxin, or critical illness. Scand J Gastroenterol. 1990;25:865‐875. 2218392 10.3109/00365529008997606

[20] Sanchez LC , Murray MJ , Merritt AM . Effect of omeprazole paste on intragastric pH in clinically normal neonatal foals. Am J Vet Res. 2004;65:1039‐1041. 15334835 10.2460/ajvr.2004.65.1039

