# Supplementary material for: Intragastric pH of foals admitted to the intensive care unit
Source: J Vet Intern Med. 2020 Sep 29;34(6):2719–26. doi: 10.1111/jvim.15888 (PMC7694801; doi:10.1111/jvim.15888)

Supporting Information Figure S1*:* Photograph of the indwelling nasogastric tube, with the calibrated disposable animony pH probe with the 2 electrodes 5 cm apart, seated within the lumen. The pH probe is secured at the most proximal end of the nasogastric tube. The pH probe is connected to a data logger that was secured to the foal's neck by a bandage.

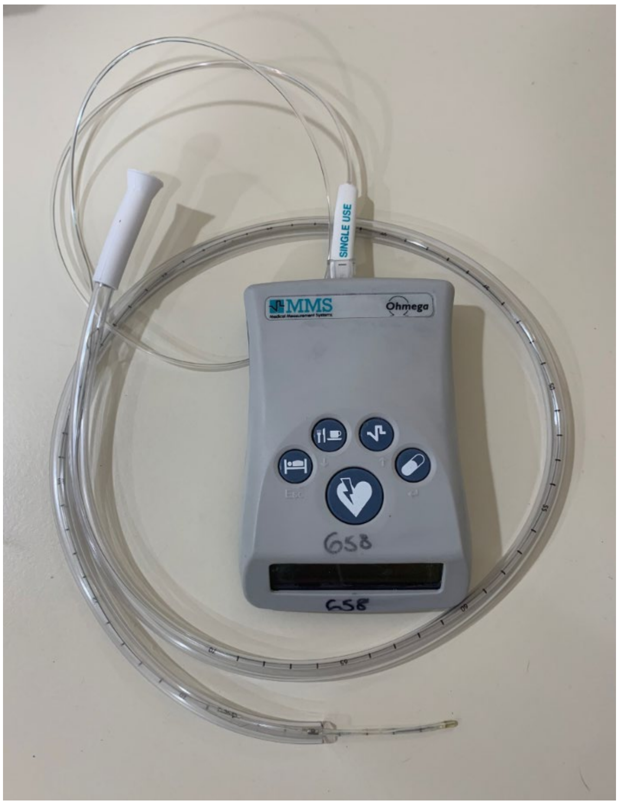

Supplement: Supplementary file 1 — Supplementary Item 1 Photograph of the indwelling nasogastric tube, with the calibrated disposable antimony pH probe with 2 electrodes 5 cm apart, seated within the lumen. The pH probe is secured at the most proximal end of the nasogastric tube. The pH probe is connected to a data logger that was secured to the foal's neck by a bandage. [file JVIM-34-2719-s001.pdf]
