# Supplementary material for: Intragastric pH of foals admitted to the intensive care unit
Source: J Vet Intern Med. 2020 Sep 29;34(6):2719–26. doi: 10.1111/jvim.15888 (PMC7694801; doi:10.1111/jvim.15888)
Supplement: Supplementary file 2 — Supplementary Item 2 Clinical data collected at the time of admission from 42 foals presented to the ICU that underwent measurement of intragastric pH. [file JVIM-34-2719-s002.docx]

**Supporting Information Table S2:** Clinical data collected at the time of admission from 42 foals presented to the ICU that underwent measurement of intragastric pH.

| **Foal** | **Sex** | **Breed** | **Gestational age (days)** | **Age at the time of admission (hours)** | **Refined Diagnosis** | **Recumbent/ Unable to stand/ Ambulatory** | **Has the foal nursed** | **Failure of passive transfer (IgG <8g/L)** | **Lactate (mmol/L)** | **PaO2 (mmHg)** | **PaCO2 (mmHg)** | **pH** | **Outcome (Discharge/ Died/ Euthanasia)** | **Positive blood culture** | **Bacteria isolated** |
| --- | --- | --- | --- | --- | --- | --- | --- | --- | --- | --- | --- | --- | --- | --- | --- |
| 1 | Colt | Anglo Arab | Unknown | 7 days | Clostridium difficile enterocolitis and Bronchopneumonia (Actinobacillus sp.) | Ambulatory | yes | no | 2.8 | 80 | 39 | 7.5 | Discharged | Yes | *Escherichia coli* |
| 2 | Filly | SB | 350 | 24 | NMS & Diarrhoea | Recumbent | no | yes |  |  |  |  | Discharged | No |  |
| 3 | Colt | American Paint | 327 | 18 | NMS | Recumbent | no | yes | 4 | 24 | 67 |  | Euthanasia | No |  |
| 4 | Colt | SB | 354 | 30 | NMS | Ambulatory | no | yes | 3.8 | 73 | 52 | 7.4 | Died | Not examined |  |
| 5 | Filly | TB | 344 | 2 | NMS | Recumbent | no | yes | 7.8 | 25 | 58 | 7.2 | Discharged | No |  |
| 6 | Colt | TB | 345 | 6 days | Clostridium difficile enterocolitis | Ambulatory | yes | yes | 1.7 | 86 | 34 | 7.2 | Discharged | No |  |
| 7 | Colt | QH | 319 | 5 | Premature & NMS | Recumbent | no | yes | 8 |  |  | 7.3 | Discharged | No |  |
| 8 | Filly | SB | 349 | 30 | NMS | Ambulatory | no | yes | 2 | 73 | 55 | 7.4 | Discharged | No |  |
| 9 | Colt | SB | 382 | 14 | NMS | Ambulatory | no | yes | 3.7 | 70 | 48 | 7.4 | Discharged | Not examined |  |
| 10 | Colt | QH | unknown | 6 | Orphan | Ambulatory | yes | no | 2.5 | 45 | 51 | 7.4 | Discharged | Not examined |  |
| 11 | Colt | TB | 365 | 12 | NMS & Diarrhoea | Ambulatory | no | yes | 3.1 | 45 | 59 | 7.4 | Discharged | No |  |
| 12 | Colt | Connamara cross | 329 | 5 | NMS | Ambulatory | yes | no | 2.4 | 52 | 44 | 7.4 | Discharged | Not examined |  |
| 13 | Colt | SB | 368 | 6 | Diarrhoea | Ambulatory | yes | no |  |  |  |  | Discharged | Not examined |  |
| 14 | Colt | American Paint | 344 | 12 | Admitted with sick mare | Ambulatory | yes | no | 1.5 | 70 | 46 | 7.4 | Discharged | Not examined |  |
| 15 | Filly | TB | 349 | 5 | NMS | Ambulatory | yes | yes | 3.6 | 58 | 65 | 7.4 | Discharged | Not examined |  |
| 16 | Colt | American paint | 336 | 4 | NMS | Ambulatory | no | yes | 1.2 | 80 | 44 | 7.4 | Discharged | No |  |
| 17 | Filly | TB | 344 | 11 days | Septic arthritis | Ambulatory | yes | yes |  |  |  |  | Discharged | Not examined |  |
| 18 | Colt | TB | 324 | 9 | NMS | Ambulatory | no | no | 4.8 | 32 | 66 |  | Discharged | No |  |
| 19 | Filly | TB | 334 | 5 | Orphan | Ambulatory | yes | no | 1.7 |  |  | 7.4 | Discharged | Not examined |  |
| 20 | Filly | SB | 346 | 36 | NMS | Ambulatory | yes | no | 7.1 | 50 | 53 | 7.3 | Discharged | Yes | *Klebsiella pneumoniae* |
| 21 | Colt | SB | 352 | 6 | NMS | Ambulatory | no | yes | 4.2 | 88 | 39 | 7.5 | Discharged | Not examined |  |
| 22 | Colt | SB | 365 | 5 | NMS & Diarrhoea | Ambulatory | yes | yes | 3.7 | 50 | 52 | 7.4 | Discharged | No |  |
| 23 | Colt | SB | 338 | 36 | NMS & Diarrhoea | Recumbent | yes | no | 14 |  |  | 7.1 | Discharged | No |  |
| 24 | Colt | TB | 343 | 4 days | Clostridium perfringens | Ambulatory | yes | no | 1.6 | 62 | 48 | 7.4 | Discharged | Yes | *Micrococcus sp.* |
| 25 | Filly | Arabian | 335 | 6 | ALD | Ambulatory | yes | no |  |  |  |  | Discharged | Not examined |  |
| 26 | Filly | Clydesdale |  | 3 | NMS | Ambulatory | no | yes | 2.2 | 69 | 51 | 7.4 | Discharged | Not examined |  |
| 27 | Filly | TB | 344 | 4 | Dysmature & NMS | Ambulatory | no | yes | 11.8 | 74 | 44 | 7.3 | Discharged | No |  |
| 28 | Filly | TB | 328 | 7 | NMS | Recumbent | no | yes | 15.8 | 59 | 46 | 7.4 | Discharged | No |  |
| 29 | Filly | TB | 312 | 8 | Premature & NMS | Ambulatory | no | yes | 2.9 |  |  | 7.6 | Euthanasia | No |  |
| 30 | Colt | TB | 335 | 24 | NMS | Recumbent | no | yes | 8 | 64 | 55 | 7.3 | Euthanasia | No |  |
| 31 | Colt | TB | 352 | 6 days | Neonatal Isoerythrolysis | Ambulatory | yes | no | 5.9 |  | 37 | 7.5 | Discharged | No |  |
| 32 | Colt | TB | 348 | 14 | NMS | Recumbent | no | yes | 3.5 | 55 | 50 | 7.3 | Discharged | No |  |
| 33 | Colt | TB | 336 | 48 | Clostridium difficile enterocolitis | Ambulatory | yes | yes | 1 | 76 | 44 | 7.3 | Discharged | Yes | *Staphylococcus sp.* |
| 34 | Colt | SB | 337 | 12 | NMS | Recumbent | no | yes | 7.6 | 31 | 57 | 7.4 | Euthanasia | Yes | *Alcaligenes faecalis* |
| 35 | Colt | TB | 343 | 16 | NMS | Ambulatory | yes | yes | 4 | 63 | 51 | 7.5 | Discharged | No |  |
| 36 | Filly | SB | 351 | 3 | NMS | Recumbent | no | yes | 15 | 85 | 25 | 7.4 | Discharged | No |  |
| 37 | Colt | Anglo Arab |  | 40 | Urachal rent | Ambulatory | yes | no | 1.8 | 65 | 50 | 7.4 | Discharged | Yes | *Enterococcus sp.* |
| 38 | Filly | SB | 345 | 2 | NMS | Recumbent | no | yes | 8.7 |  |  |  | Euthanasia | Yes | *Streptococcus sp* |
| 39 | Colt | TB | 306 | 7 | Premature & NMS | Recumbent | no | yes | 1.9 | 66 | 51 | 7.4 | Euthanasia | Yes | *Cellulosimicrobium cellulans* |
| 40 | Colt | QH | 339 | 0 | NMS | Recumbent | no | yes | 15 |  |  | 6.8 | Euthanasia | No |  |
| 41 | Colt | SB | 341 | 18 | NMS & Diarrhoea | Recumbent | no | yes | 4.9 | 32 | 62 | 7.3 | Discharged | No |  |
| 42 | Filly | Friesian | 316 | 18 | Diarrhoea & Hepatopathy | Ambulatory | yes | no | 4.6 | 47 | 63 | 7.5 | Discharged | Yes | *Staphylococcus aureus* |

NMS, neonatal maladjustment syndrome; SB, Standardbred; TB, Thoroughbred; QH, Quarter horse
