# Supplementary material for: Intragastric pH of foals admitted to the intensive care unit
Source: J Vet Intern Med. 2020 Sep 29;34(6):2719–26. doi: 10.1111/jvim.15888 (PMC7694801; doi:10.1111/jvim.15888)
Supplement: Supplementary file 3 — Supplementary Item 3 Intragastric pH data collected from 42 foals that were presented to the ICU. [file JVIM-34-2719-s003.pdf]

Supporting Information Table S3: Intragastric pH data collected from 42 foals that were presented to the ICU.

Foal	Recording Duration	Proximal Electrode Mean pH	Min pH	Max pH	% time <pH 4	Distal Electrode Mean pH	Min pH	Max pH	% time <pH 4
1	28.02	6.2	0.4	8.7	22.56	7.3	2.2	8.4	7.0
2	26.41	6.5	0	14	4.48	6.6	0	10	3.4
3	26.73	5.6	0.2	14	6.72	6.7	1.2	10	4.5
4	4.82	5.2	0	14	7.81	4.2	0.8	8.4	19.7
5	4.99	5.1	0.4	12.6	13.00	4.6	0	10	22.0
6	36.19	0.9	0	13.3	57.48	2.3	0	9.9	52.8
7	19.69	7.2	1.2	14	0.01	7.4	2.2	10	0.0
8	24.94	6.7	0	14	1.97	5.0	0	10	32.6
9	19.15	5.5	0.6	14	12.96	4.2	0	10	25.1
10	26.34	7.7	4.7	11.1	0.00	7.8	4.6	9	0.0
11	10.38	7.1	0	14	1.37	6.9	0	10	0.2
12	30.46	5.6	0	14	5.46	5.2	0	10	6.6
13	21.60	4.4	0	10.9	45.83	5.5	0	10	27.1
14	2.12	1.9	0.1	13	99.65	1.2	0	9.8	81.6
15	7.08	7.8	6.2	9.4	0.00	7.4	4.9	7.2	0.0
16	0.66	6.1	5.8	13.4	0.00	6.3	5.9	6.8	0.0
17	14.23	5.9	0.3	14	0.54	5.2	0.4	10	9.2
18	35.99	6.7	3.6	10.8	0.54	7.5	3.6	10	0.0
19	18.32	7.8	0	14	0.16	7.9	0.9	9.7	1.4
20	9.05	5.8	3.3	10.1	3.16	6.0	2.9	10	11.0
21	2.75	2.6	0	12.3	95.00	1.6	0	10	18.8
22	17.43	6.0	0	14	7.21				
23	38.92	4.8	0.1	8	28.83	3.7	0	7.7	52.6
24	25.41	4.4	0	14	35.10	4.8	0	10	43.5
25	40.69	7.2	2.1	12.3	0.00	6.6	1.2	9.6	5.1
26	22.67	6.5	2.6	13	4.63	7.0	1.9	9.9	6.8
27	25.81	2.5	0	13.6	80.80	2.2	0	9.9	81.4
28	23.59	5.3	0.6	13.3	13.07	2.8	0	9.7	73.4
29	12.74	4.3	1.2	13.3	26.63	2.7	0.4	10	81.4
30	8.14	5.9	0.7	14	0.11	6.1	0.1	10	2.4
31	22.47	5.2	0.1	14	16.56	4.1	0.8	10	46.7

32	25.26	7.3	0	11.4	0.09	6.5	0.1	9.2	10.8
33	9.15	4.9	0.4	9	19.79	4.8	0	9	18.1
34	9.84	5.1	1.4	6.4	0.52	4.8	0.1	6.2	9.7
35	3.93	4.4	0.6	13.7	37.22	5.3	0	10	18.5
36	21.48	3.2	0.9	6.2	75.46	3.9	0	8.2	52.6
37	3.29	6.3	0.6	10.2	0.05	5.8	3.9	9.3	0.0
38	23.22	6.1	2.2	10.8	5.36	5.8	1.2	9.3	6.8
39	30.76	5.9	1.5	14	5.85	3.7	0	10	61.2
40	12.61	11.3	0	14	0.10	8.4	0	10	0.1
41	22.91	4.1	0	12.8	49.43	3.8	0	9.8	51.2
42	15.92	4.1	0	14	31.23	3.1	0	10	45.6
