# Supplementary material for: Intragastric pH of foals admitted to the intensive care unit
Source: J Vet Intern Med. 2020 Sep 29;34(6):2719–26. doi: 10.1111/jvim.15888 (PMC7694801; doi:10.1111/jvim.15888)
Supplement: Supplementary file 4 — Supplementary Item 4 Results of univariate analyses of associations between dichotomised clinical parameters (yes/no) and the outcome variables of mean pH and % time < pH 4, for both the proximal and distal electrodes. [file JVIM-34-2719-s004.pdf]

**Supporting Information Table S4:** Results of univariate analyses of associations between dichotomised clinical parameters (yes/no) and the outcome variables of mean pH and % time < pH 4, for both the proximal and distal electrodes.

| ***Clinical parameters collected at time of presentation (yes/no)*** | **Mean pH** | | **% time pH < 4** | |
|---|---|---|---|---|
| | **Proximal electrode** | **Distal electrode** | **Proximal electrode** | **Distal electrode** |
| | **P-value** | **P-value** | **P-value** | **P-value** |
| Acidosis | 0.69 | 0.71 | 0.94 | 0.42 |
| Ambulatory | 0.43 | 0.44 | 0.38 | 0.34 |
| Diarrhoea | 0.15 | 0.52 | 0.02 | 0.23 |
| Dystocia | 0.72 | 0.67 | 1.00 | 0.96 |
| Mare with evidence of placentitis | 0.002 | 0.002 | 0.01 | 0.02 |
| Filly | 0.63 | 0.86 | 0.68 | 0.49 |
| Hyperlactataemia | 0.73 | 0.49 | 0.44 | 0.57 |
| Hypoxia | 0.96 | 0.91 | 0.70 | 0.93 |
| Nursing | 0.58 | 0.94 | 0.59 | 0.99 |
| NMS | 0.43 | 0.85 | 0.58 | 0.92 |
| Premature | 0.87 | 0.4 | 0.88 | 0.21 |
| Sepsis | 0.83 | 0.8777 | 0.90 | 0.79 |
| Survival | 0.41 | 0.8396 | 0.49 | 0.99 |
| < 24 hours of age at presentation | 0.51 | 0.5767 | 0.56 | 0.40 |
