# Supplementary material for: Intragastric pH of foals admitted to the intensive care unit
Source: J Vet Intern Med. 2020 Sep 29;34(6):2719–26. doi: 10.1111/jvim.15888 (PMC7694801; doi:10.1111/jvim.15888)
Supplement: Supplementary file 5 — Supplementary Item 5 Results of univariate analyses of associations between clinical parameters (yes/no) and pH > 4 for >50% of the recording period (pH >4>50) and pH >4 for >80% of the recording period (pH >4>80) as dichotomous variables (yes/no) for both the proximal and distal electrodes. [file JVIM-34-2719-s005.pdf]

**Supporting Information Table S5:** Results of univariate analyses of associations between clinical parameters (yes/no) and pH > 4 for >50% of the recording period (pH > $4_{>50}$) and pH >4 for >80% of the recording period (pH >$4_{>80}$) as dichotomous variables (yes/no) for both the proximal and distal electrodes.

| ***Clinical parameters collected at time of presentation*** | **pH > $4_{>50}$** | | | | | | **pH >$4_{>80}$** | | | | | |
|---|---|---|---|---|---|---|---|---|---|---|---|---|
| | **Proximal electrode** | | | **Distal electrode** | | | **Proximal electrode** | | | **Distal electrode** | | |
| | **OR** | **95% CI** | **P-value** | **OR** | **95%CI** | **P-value** | **OR** | **95%CI** | **P-value** | **OR** | **95%CI** | **P-value** |
| <24hours old | 0.6 | 0.01-7.01 | 0.65 | 0.64 | 0.06-4.23 | 0.7 | 1.16 | 0.2-5.87 | 0.83 | 1.16 | 0.23-5.58 | 0.82 |
| Acidosis | 0.81 | 0.08-10.9 | 0.61 | 0.37 | 0.06-2.15 | 0.25 | 0.85 | 0.18-4.2 | 0.80 | 0.61 | 0.14-2.7 | 0.52 |
| Ambulatory | 1.77 | 0.13-18 | 0.74 | 0.64 | 0.06-4.23 | 0.7 | 0.35 | 0.03-2.14 | 0.28 | 0.42 | 0.06-2.16 | 0.31 |
| Diarrhoea | 1.47 | 0.12-80. | 0.61 | 0.57 | 0.09-4.41 | 0.66 | 0.15 | 0.02-0.78 | 0.02 | 0.33 | 0.05-1.74 | 0.15 |
| Dystocia | 0.56 | 0.06-7.69 | 0.57 | 0.67 | 0.11-5.13 | 0.68 | 1.49 | 0.28-10 | 0.72 | 0.7 | 0.14-3.64 | 0.72 |
| Evidence of placentitis | n/a | n/a | 0.83 | n/a | n/a | 0.31 | n/a | n/a | 0.08 | n/a | n/a | 0.07 |
| Filly | 0.82 | 0.08-10.95 | 0.80 | 0.66 | 0.11-4.05 | 0.7 | 1.36 | 0.29-7.58 | 0.74 | 0.61 | 0.14-2.7 | 0.52 |
| Hyperlactataemia | 0.79 | 0.06-7.75 | 0.64 | 0.91 | 0.15-5.13 | 0.9 | 1.05 | 0.23-4.74 | 0.94 | 0.85 | 0.2-3.53 | 0.79 |
| Hypoxia | 1.94 | 0.2-25.79 | 0.83 | 0.91 | 0.15-5.13 | 0.9 | 1.63 | 0.36-7.6 | 0.52 | 0.85 | 0.2-3.53 | 0.79 |
| NMS | 1.38 | 0.1-13.78 | 0.74 | 0.48 | 0.04-3.15 | 0.69 | 2.2 | 0.46-10.7 | 0.30 | 1.27 | 0.27-5.7 | 0.75 |

| | | | | | | | | | | | | |
|---|---|---|---|---|---|---|---|---|---|---|---|---|
| Nursing | 1.27 | 0.13-17 | 0.80 | 1.74 | 0.3-12.67 | 0.71 | 0.39 | 0.08-1.77 | 0.19 | 1 | 0.24-4.34 | 0.99 |
| Premature | n/a | n/a | 0.44 | 0.24 | 0.02-3.91 | 0.2 | 0.42 | 0.03-6.43 | 0.58 | 0.19 | 0.003-2.62 | 0.28 |
| Sepsis | n/a | n/a | 0.57 | 2.61 | 0.27-132.8 | 0.65 | 0.87 | 0.15-6.47 | 0.86 | 1.36 | 0.24-9.92 | 0.69 |
| Survival | 0 | 0-4.86 | 0.56 | 1.23 | 0.1-9.2 | 0.82 | 0.27 | 0.005-2.52 | 0.40 | 0.46 | 0.04-3.1 | 0.45 |
