# Supplementary material for: Intragastric pH of foals admitted to the intensive care unit
Source: J Vet Intern Med. 2020 Sep 29;34(6):2719–26. doi: 10.1111/jvim.15888 (PMC7694801; doi:10.1111/jvim.15888)
Supplement: Supplementary file 6 — Supplementary Item 6 Results of univariate logistic regression analyses of associations between continuous clinical parameters and the outcome variables pH >4 for >50% of the recording period (pH >4>50) and pH >4 for >80% of the recording period (pH >4>80). [file JVIM-34-2719-s006.pdf]

Supporting Information Table S6: Results of univariate logistic regression analyses of associations between continuous clinical parameters and the outcome variables pH > 4 for >50% of the recording period (pH > $4_{>50}$) and pH >4 for >80% of the recording period (pH >$4_{>80}$).

Clinical parameters collected at the time of presentation	**pH > $4_{>50}$**						**pH > $4_{>80}$**					
	**Proximal electrode**			**Distal electrode**			**Proximal electrode**			**Distal electrode**		
	**OR**	**95% CI**	**P-value**	**OR**	**95%CI**	**P-value**	**OR**	**95%CI**	**P-value**	**OR**	**95%CI**	**P-value**
Age at the time of admission	1	0.94-312	0.91	1	0.99-1.02	0.73	1	0.98-1	0.44	1	0.99-1.01	0.82
Albumin	1.11	0.87-1.43	0.4	1.24	0.99-1.55	0.06	1.05	0.91-1.24	0.5	1.05	0.9-1.21	0.56
Blood glucose	1.24	0.83-1.85	0.29	1.12	0.86-1.46	0.39	0.85	0.66-1.04	0.14			0.58
Calcium	16.3	0.35-771.71	0.16	7.69	0.26-229.57	0.24	4.82	0.33-194.64	0.32	6.03	0.27-137	0.26
Chloride	0.91	0.78-1.05	0.19	1.02	0.91-1.15	0.75	0.86	0.72-9.77	0.05	0.92	0.82-1.03	0.17
Creatinine	0.998	0.996-0.999	0.04	1	1-1.002	0.84	1	1-1.001	0.54	1	0.999-1.002	0.55

Fibrinogen	3.23	0.57-18.27	0.18	0.81	0.5-1.32	0.39	0.89	0.56-1.44	0.62	0.88	0.57-1.36	0.57
Gestational age	0.97	0.91-1.03	0.36	1.04	9.86-1.1	0.14	1	0.95-1.05	0.99	0.99	0.95-1.04	0.82
Globulins	1.1	0.92-1.33	0.27	1.03	0.92-1.15	0.63	1	0.92-1.11	0.86	1	0.92-1.09	0.96
Haematocrit	12.49	0.0002-958220.1	0.66	0.3	0-738.45	0.76	0.39	0.0003 -533.83	0.79	56	0.02-126865	0.31
Heart rate	1.03	0.98-1.08	0.33	1.02	0.98-1.06	0.33	1.01	0.98-1.05	0.40	1	0.98-1.04	0.51
MAP	0.93	0.822-1.05	0.24	0.95	0.86-1.05	0.29	0.99	0.91-1.07	0.74	1	0.94-1.08	0.82
Neutrophil concentration	1.03	0.21-0.29	0.78	0.86	0.71-1.03	0.09	0.97	0.84-1.12	0.64	0.87	0.73-1.04	0.14
PaCO2	1.31	1.04-1.66	0.02	1.1	0.99-1.22	0.08	1.1	1-1.23	0.06	1.05	0.97-1.14	0.2

PaO2	0.81	0.67-9.73	0.03	1	0.91-1.02	0.23	0.95	0.89-1	0.07	0.99	0.95-1.03	0.55
pH	0.92	5.39-1.57	0.98	4.2	1.67-1.05	0.61	0.2	0.19-5.03	0.6	0.67	3.44-1.28	0.88
RBC	1.15	0.74-1.8	0.54	0.99	0.73-1.36	0.96	0.93	0.7-1.23	0.61	1.13	0.85-1.51	0.4
Respiratory rate	1.01	0.96-1.06	0.77	1	0.96-1.03	0.77	0.99	0.96-1.03	0.66	0.97	0.94-1	0.06
SAA	1	0.995-1	0.65	1	0.997-1.01	0.38	1	1-1.01	0.19	1	0.998-1.007	0.29
Systemic lactate concentration	0.91	0.75-1.11	0.37	0.85	0.71-1	0.06	0.96	0.81-1.14	0.62	0.9	0.77-1.06	0.22
Temperature	1.01	0.92-1.11	0.79	1.1	0.78-1.53	0.59	1.02		0.63	1.03	0.92-1.15	0.61
Total protein	1.11	0.94-1.31	0.22	1.1	0.96-1.19	0.2	1.02	0.95-1.1	0.64	1.01	0.94-1.09	0.75

Urea	0.98	0.76-1.27	0.90	1.1	0.84-1.32	0.64	1.03	0.86-1.25	0.78	1.04	0.87-1.24	0.69
WBC	1	0.85-1.35	0.98	0.86	0.71-1.03	0.1	0.94	0.83-1.07	0.37	0.88	0.74-1.05	0.16
Weight	0.95	0.86-1.05	0.36	1.03	0.97-1.11	0.34	1	0.93-1.05	0.72	0.99	0.94-1.05	0.75
Sodium	1.03	0.009-1.19	0.69	0.94	0.84-1.04	0.22	0.99	0.89-1.09	0.77	0.95	0.87-1.05	0.32
Potassium	1.24	0.38-4.03	0.72	1.49	0.53-4.2	0.45	1.35	0.65-3.77	0.5	1.38	0.62-3.05	0.43
