# Supplementary material for: Intragastric pH of foals admitted to the intensive care unit
Source: J Vet Intern Med. 2020 Sep 29;34(6):2719–26. doi: 10.1111/jvim.15888 (PMC7694801; doi:10.1111/jvim.15888)
Supplement: Supplementary file 7 — Supplementary Item 7 Comparison of the distal electrode in the first recording period to the second recording period. There is no significant difference in mean pH or % time pH < 4 in the 2 recording periods. [file JVIM-34-2719-s007.pdf]

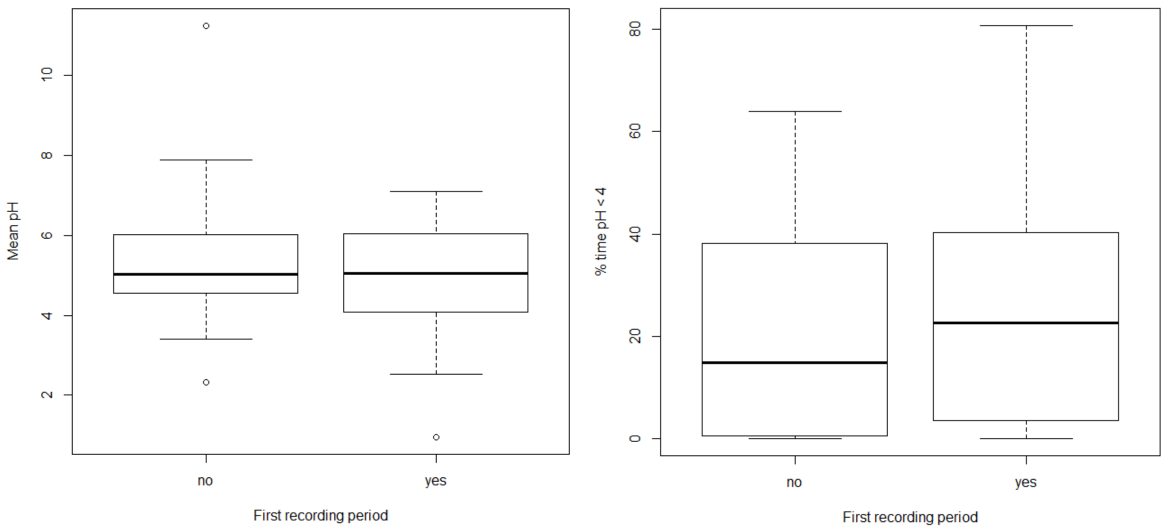

Supporting Information Figure S7: Comparison of the distal electrode in the first recording period to the second recording period. There is no difference in mean pH or % time pH < 4 in the 2 recording periods.
